# NMR Metabolomics and Random Forests Models to Identify Potential Plasma Biomarkers of Blood Stasis Syndrome With Coronary Heart Disease Patients

**DOI:** 10.3389/fphys.2019.01109

**Published:** 2019-09-04

**Authors:** Lin-Lin Zhao, Xin-Jian Qiu, Wen-Bo Wang, Ruo-Meng Li, Dong-Sheng Wang

**Affiliations:** ^1^Health Management Department, The Third Xiangya Hospital, Central South University, Changsha, China; ^2^Institute of Integrated Traditional Chinese and Western Medicine, Xiangya Hospital, Central South University, Changsha, China; ^3^Traditional Chinese Medicine Department, The Third Xiangya Hospital, Central South University, Changsha, China

**Keywords:** coronary heart disease, blood stasis syndrome, metabolomics, random forests, ZHENG types, Systems Biology

## Abstract

**Background:**

Coronary heart disease (CHD) remains highly prevalent and is one of the largest causes of death worldwide. Blood stasis syndrome (BSS) is the main syndrome associated with CHD. However, the underlying biological basis of BSS with CHD is not yet been fully understood.

**Materials and Methods:**

We proposed a metabolomics method based on ^1^H-NMR and random forest (RF) models to elucidate the underlying biological basis of BSS with CHD. Firstly, 58 cases of CHD patients, including 27 BSS and 31 phlegm syndrome (PS), and 26 volunteers were recruited from Xiangya Hospital affiliated to Central South University. A 1 mL venous blood sample was collected for NMR analysis. Secondly, principal component analysis (PCA), partial least squares discrimination analysis (PLS-DA) and RF was applied to observe the classification of each group, respectively. Finally, RF and multidimensional scaling (MDS) were utilized to discover the plasma potential biomarkers in CHD patients and CHD–BSS patients.

**Results:**

The models constructed by RF could visually discriminate BSS from PS in CHD patients. Simultaneously, we obtained 12 characteristic metabolites, including lysine, glutamine, taurine, tyrosine, phenylalanine, histidine, lipid, citrate, choline, lactate, α-glucose, β-glucose related to the CHD patients, and Choline, β-glucose, α-glucose and tyrosine were considered as potential biomarkers of CHD–BSS.

**Conclusion:**

The combining of ^1^H-NMR profiling with RF models was a useful approach to analyze complex metabolomics data (should be deleted). Choline, β-glucose, α-glucose and tyrosine were considered as potential biomarkers of CHD–BSS.

## Background

Coronary heart disease (CHD) is always associated with metabolic disorder, and the metabolic risk factors, such as hyperlipidemia and diabetes, powerfully predict the cardiac events (Lacoste et al., 1995; Ansar et al., 2011). CHD remains highly prevalent and is one of the largest causes of death worldwide. Specific and sensitive diagnostic methods are critical both for early detection and treatment of CHD (Rubin, 2013). Metabonomics technologies have been studied to diagnose the presence and severity of CHD (Brindle et al., 2002). It is a newly developed platform of systems biology that allows holistic, quantitative and qualitative determination of low molecular endogenous metabolites in biofluids or tissues (Lindon et al., 2004). It concerns the dynamic multivariate metabolic changes in response to pathophysiological stimuli, genetic modification, environmental influences or drug perturbations (Nicholson and Lindon, 2008). Analysis of differential metabolites enables us to obtain novel biomarkers discovery and disturbed metabolic pathways (Sabatine et al., 2005).

Nowadays, Traditional Chinese Medicine (TCM) has become popular worldwide. Systematic, holistic and dynamic characteristics of TCM theory are perfectly coincident with metabonomics (Wang et al., 2005; Sun et al., 2012). Metabonomics technologies have been applied in study on the essence of the syndrome (ZHENG type or pattern in TCM) and shown the superiority and advanced nature. According to TCM theory, a syndrome is a combination of clinical manifestations including symptoms, signs, tongue appearances and pulse feelings. Syndrome is not only the core of TCM theory, but also the base of definite diagnosis and effective therapies (Xu and Chen, 2008). It will not only improve the validity and reliability of “syndrome differentiation” through syndrome model established based on metabonomics, but also help to establish the clinical curative criteria.

Blood stasis syndrome (BSS) is the most common syndrome associated with CHD. Our previous study has shown that the metabonomics approach based on liquid chromatography/quadrupole time-of-flight mass spectrometry (LC-Q-TOF/MS) was useful for interpreting the differentiation of syndrome [phlegm syndrome (PS) and BSS] in TCM (Zhao et al., 2014). There were 18 differential metabolites mainly involved in amino acid metabolism, purine metabolism and pyrimidine metabolism contributing to the clustering and discrimination between PS and BSS in CHD patients. However, it is clear that the two metabonomics technologies are complementary, giving information on different sets of biomarkers and providing more comprehensive classification and biomarker information.

Random forests (RFs) is machine learning algorithm which uses an ensemble of classification trees and is an ideal method for classification and feature selection. Spectral buckets are employed as input variables. RF could be employed for both supervised (outcome labels are used) and unsupervised (outcome labels are not used) learning (Breiman, 2001). In this study, we proposed a metabolomics method based on ^1^H-NMR and RF models to validate the separated trend between BSS and PS in CHD patients and acquire more potential biomarkers of BSS in CHD patients.

## Materials and Methods

### Subjects Collection

A total of 58 cases CHD patients (27 BSS and 31 PS) and 26 volunteers were derived from the Xiangya Hospital affiliated to the Central South University in Hunan Province, China (June 1, 2013 to April 30, 2014). All selected CHD patients were diagnosed and confirmed by coronary angiography. Diagnosis standard of CHD refers to “nomenclature and diagnosis criteria of ischemic heart disease” which is established by the Joint International Society and Federation of Cardiology/World Health Organization Task Force on Standardization of Clinical Nomenclature (Nomenclature and criteria for diagnosis of ischemic heart disease, 1979). The syndrome was identified by three chief physicians, according to “criteria for TCM syndrome differentiation of patients with coronary heart disease” (Subcommittee of Cardiovascular Diseases of China Society of Integrated Traditional Chinese, and Western Medicine, 1991).

Patients who suffered from diabetic cardiomyopathy, hyperthyroid heart disease, hypertensive heart disease, pulmonary heart disease, anemic heart disease, systemics scleroderma heart disease, inborn coronary abnormity and rheumatic heart disease, who suffered from severe hypertension, malignant tumor, renal failure, thyroid disease, pulmonary infection, who suffered from infectious diseases, who suffered from invigorative system disease and women in pregnant or in lactation were excluded.

All patients aged from 45 to 75 were eligible for enrollment. The age and sex of volunteers in control group were group-matched with the cases group. The study was approved by the hospital ethics committee and all subjects provided written informed consent.

### Chemicals and Reagents

NaCl, K_2_HPO_4_⋅3H_2_O, and NaH_2_PO_4_⋅2H_2_O (all in analytical-grade) were obtained from Sinopharm Chemical Reagent Co., Ltd. (Shanghai, China); NaN_3_ (in analytical-grade, used for anticorrosion) was provided by the Tianjin Fu Chen Chemical Reagent Factory. D_2_O (in analytical-grade) was produced from Cambridge Isotope Laboratories Inc., United States.

### Plasma Collection and Preparation

A 1 mL venous blood sample was collected in a vacutainer tubes with ethylenediaminetetraacetic acid (EDTA) at 7 o’ clock in the morning after 12 h of overnight fasting and then centrifuged at 10,000 rpm for 10 min at 4°C. The supernatant was stored at −80°C until NMR analysis.

A total of 200 μL plasma was mixed with 400 μL of K_2_HPO_4_/NaH_2_PO_4_ buffer (45 mM, pH 7.4) containing 50% D_2_O and 0.9% NaCl in a centrifuge tube (1.5 mL), after centrifugation (12,000 rpm) for 10 min, a total of 550 μL of the supernatant was placed into a 5 mm NMR tubes directly for NMR analysis.

### NMR Analysis

All ^1^H NMR spectra were measured at 298 K on a Bruker AVIII 600 spectrometer (Bruker Biospin, Germany) equipped with Ultra cryogenic probe operating at 600.13 MHz for ^1^H.

A standard one-dimensional (1D) ^1^H-NMR spectra were recorded using a NOESYPR1D pulse sequence [recycle delay (RD)-90°-*t*_1_-90°-*t*_m_-90°-acquire] (Nicholson et al., 1995) to obtain all observed metabolites signals, CPMG pulse sequence [RD-90°-(τ-180°-τ)_n_-acquire] (Meiboom and Gill, 1958) to obtain small molecule metabolites signals and DIFFUSIONEDIT [RD-90°-G_1_-τ-180°-G_2_-τ-90°-△-90°-G_3_-τ-180°-G_4_-τ-90°-Te-90°- acquire] to obtain macromolecules metabolites. The following parameters were set for NMR detection in the experiments: 90° pulse length (*P*_1_) was 11.2 μs, PLW_1_ was 10, −10 [W, −dBW], spectral width was 20 ppm, the sampling point was 32 k, the acquisition time was 1.64 s. 64 scans, 8 dummy scans. In the NOESYPR1D experiments, *t*_1_ was 4 μs, mixing time (D_8_) was 100 ms, relaxation decay time (D_1_) was 2 s, In the CPMG experiments, total echo time: 80 ms, Echo evolution time (d20): 350 μs, Echo cycle (L_4_): 100. In the DIFFUSIONEDIT experiments, gradient recovery (D_16_): 150 μs, diffusion time (D_20_): 0.2 s; Gradient strength (GPZ6) 85%, gradient pulse length (P_30_): 1100 μs.

For resonance assignment, a range of two-dimensional (2D) NMR spectra were acquired including ^1^H-^1^H correlation spectroscopy (COSY), ^1^H-^1^H total correlation spectroscopy (TOCSY) NMR spectra,^1^H-^1^H J-Resolved Spectroscopy (JRES), ^1^H-^13^C heteronuclear single quantum correlation (HSQC) and ^1^H-^13^C heteronuclear multiple bond correlation spectra (HMBC). Briefly, in COSY and TOCSY experiments, the sampling point of 160 (*F*_1_) and 2048 (*F*_2_), spectral width of 10.5 ppm (*F*_1_) and 10.5 ppm (*F*_2_), 90°pulse length (*P*_1_) of 11.87 s. TOCSY 2D NMR spectra were acquired with MLEV as the spin lock pulse and the spin-lock time of 80 ms. ^1^H-^1^H JRES were acquired with the sampling point of 64 (*F*_1_) and 2048 (*F*_2_) the spectral widths were 0.1 ppm in the *F*_1_ dimension and 10.5 ppm in the *F*_2_ dimension. ^1^H-^13^C HSQC 2D NMR spectra were acquired using composite pulse broad band decoupling (globally alternating optimized rectangular pulses, GARP), the sampling points were 128 (*F*_1_) and 2048 (*F*_2_), with the spectral widths of 220 ppm (*F*_1_) and 10.5 ppm (*F*_2_). ^1^H-^13^C HMBC 2D NMR spectra were acquired into 128 data points (*F*_1_) and 2048 data points (*F*_2_), the spectral widths were 220 ppm (*F*_1_) and 10.5 ppm (*F*_2_).

### Data Pre-processing of NMR Spectra and Multivariate Data Analysis

Baseline and phase corrections for the NMR spectra were manually achieved using mestrenova (version 9.0.1, Mestrelab Research, Santiago de Compostela, Spain). The peak of L-lactate with a chemical shift at δ1.33 was used as a spectral reference for plasma. The spectral region of δ0.5–9.5 was segmented into 0.002 ppm (He et al., 2009) chemical shift buckets, the region at δ4.70–5.00 and δ 5.6–6.0 were discarded to eliminate the effects of imperfect water suppression and urea signal, respectively. The regions of EDTA-Ca resonance (δ2.55–2.58, δ3.07–3.17), EDTA-Mg resonance (δ2.68–2.70, δ3.23–3.26), Free-EDTA resonance (δ3.6–3.63) were removed. The integral value of each spectrum was used as input variables for the subsequent statistical analysis.

Principal component analysis (PCA) and partial least squares discrimination analysis (PLS-DA) were performed to examine intrinsic variation of the NMR spectral data using SIMCA-P 14.0 (Umetrics, Sweden). Each point on the scores plot is defined by the spectrum of an individual sample. Then, RFs in software MATLAB (2010b, The MathWorks Inc., Natick, MA, United States) were applied to uncover the underlying structure of this data, multidimensional scaling (MDS) was employed to map the proximity into a lower-dimensional space.

A one-way analyses of variance (ANOVA) with a Bonferroni correction of the SPSS 17.0 for Windows (SPSS Inc., Chicago, IL, United States) was used for significance analysis. *P*-values less than 0.05 were considered significant.

### Random Forest

There are two powerful machine learning techniques advantages with RF: bagging and random feature selection. In bagging, each tree is trained on a bootstrap sample of the training data, and predictions are made by majority vote of trees. Instead of using all features, RF randomly selects a subset of features to split at each node when growing a tree. To assess the prediction performance of the RF algorithm, it performs a type of cross-validation in parallel with the training step by using the so-called out-of-bag (OOB) samples. On average, each tree is grown using about 2/3 of the training data, leaving about 1/3 as OOB. The RF algorithm can be stated as follows (Huang et al., 2013):

Draw *n*_tree_ bootstrap samples from the original data, *n*_tree_ is the number of ensemble tree in RF. The *n*_tree_ is equal to 2000 in this study.

For all bootstrap samples, grow an un-pruned classification or regression tree, with the following modification: at each node, rather than choosing the best split among all variables, randomly sample mtry of the variables and choose the best split from among those variables (bagging can be thought of as the special case of RFs obtained when mtry = *p*, the number of variables). In general, mtry is simply a number (positive integer) between 1 and *p*.

Predict new data by aggregating the predictions of *n*_tree_ (i.e., majority votes for classification).

#### Variable Importance

Random forest algorithm has the ability to estimate feature importance. A measure of how each feature contributes to the prediction performance of RF algorithm can be calculated in the course of the training. The important scores can be used to identify biomarkers. The frequently used type of RF algorithm to measure feature importance is the mean decrease in classification based on permutation. The prediction accuracy after permutation is subtracted from the prediction accuracy before permutation and averaged over all trees in the forest to give the permutation importance value. In the current research, the mean decrease in classification accuracy was accepted to measure variable importance.

#### Proximity Measure

Proximity matrix is the important feature of RF algorithm which can be used to identify structure in the data. RF algorithm not only generates variable-related information such as variable importance measures, but also calculates the proximities between samples. All samples in the original data set are classified by the forest. The proximity between two samples is calculated as the number of times the two samples end up in the same terminal node of a tree, divided by the number of trees in the forest. The resulting matrix is symmetric with diagonal element equal to 1 and off-diagonal elements ranging from 0 to 1. The proximities between similar samples are always high. Proximity scores may also be used to construct MDS plots. MDS plots aim to visualize the similarity or dissimilarity (calculated as 1 proximity) between samples.

## Results

### Demographic and Clinical Characteristics of the Samples

Demographic and clinical data of the subjects were summarized in Table 1. There was no significant difference in age, gender ratio, body mass index and other clinical feature between the three groups (*P* > 0.05), as assessed by Friedman’s ANOVA.

**TABLE 1:** Demographic and clinical characteristics of the samples.

	**HC**	**CHD–BSS**	**CHD–PS**	
	**(*n* = 26)**	**(*n* = 27)**	**(*n* = 31)**	***P*-value**
Age, mean ± SD, years	53.73 ± 11.39	53.70 ± 13.20	54.97 ± 10.88	0.896
Male, *n* (%)	20 (76.92)	19 (70.37)	18 (58.06)	0.298
Body mass index, mean ± SD (kg/m^2^)	24.42 ± 2.57	25.59 ± 3.89	23.61 ± 2.58	0.054
Active smoking, *n* (%)	10 (38.46)	11 (40.74)	10 (32.26)	0.785
Diabetes mellitus, *n* (%)	5 (19.23)	9 (33.33)	10 (32.26)	0.445
Hypertension, *n* (%)	8 (30.77)	9 (33.33)	11 (35.48)	0.932
Dyslipidemia, *n* (%)	7 (26.92)	14 (51.85)	16 (51.61)	0.106

### ^1^H NMR Spectrum of Plasma Samples

**FIGURE 1:**
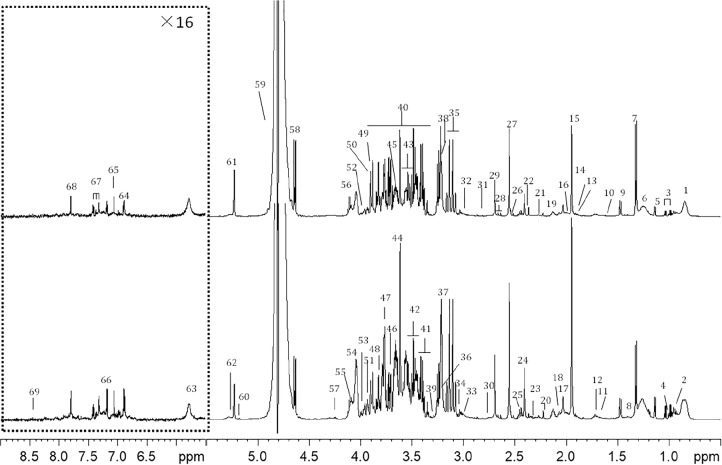
Typical cpmgpr1d spectra (600 MHz) of **(A)** CHD patient, **(B)** control from plasma samples (the region at δ5.5–9.0 was expanded for 16 times). Key: 1, lipid (CH_3_); 2, leucine and isoleucine; 3, valine; 4. isoleucine; 5, 3-hydroxybutyrate; 6, lipid (CH_2_); 7, lactate; 8, lysine; 9, alanine; 10, lipid (CH_2_CH_2_CO); 11, lysine and arginine; 12, leucine; 13, lysine and arginine; 14, acetate; 15, acetic acid (CH_3_COOH); 16, lipid (CH_2_C=C); 17, N-acetyl glycoproteins (NAG) 18, glutamate; 19, glutamine; 20, acetoacetate; 21, lipid (CH_2_C = O); 22, pyruvate; 23, glutamate; 24, succinate; 25, glutamine; 26, citrate; 27, Ca-EDTA; 28, citrate; 29, Mg-EDTA; 30, lipid (C=CCH_2_C=C); 31, trimethylamine; 32, lysine; 33, creatine; 34, creatinine; 35, Ca-EDTA; 36, choline and GPC, glycerophosphocholine (GPC) and phosphocholine (PC); 37, free-EDTA; 38, Mg-EDTA; 39, taurine; 40, glucose and amino acid; 41, α-glucose and taurine; 42, β-glucose; 43, α-glucose; 44, free-EDTA; 45, choline; 46, α-glucose; 47, β-glucose; 48, α-glucose; 49, α-glucose; 50, β-glucose; 51, tyrosine; 52, phenylalanine; 53, histidine; 54, choline; 55, TG; 56, lactate; 57, TG; 58, β-glucose; 59, H_2_O; 60, TG; 61, α-glucose; 62, lipid (CH = CH); 63, urea; 64, tyrosine; 65, histidine; 66, tyrosine; 67, phenylalanine; 68, histidine; 69, formate.

A typical 1D ^1^H NMR CMPG spectrum of plasma samples from (A) control and (B) CHD group were shown in Figure 1. Resonance assignments of metabolites were made according to the literature and confirmed by 2D NMR spectra. 69 metabolites were identified, covering amino acids, organic acids, lipids, glucose, salts, choline and urea. In addition, Tricarboxylic acid (TCA) cycle metabolites, such as succinate and citrate were included. Visual inspection of the 1D ^1^H NMR CMPG spectra showed clear differences in overall composition between CHD patients and control (Figure 1). However, these qualitative observations were all by visual inspection, multivariate data analysis of NMR spectra were performed to recover the characteristics of metabolic patterns of CHD (included BSS and PS) and control.

### Classification Using PCA, PLS-DA, and RF

**FIGURE 2:**
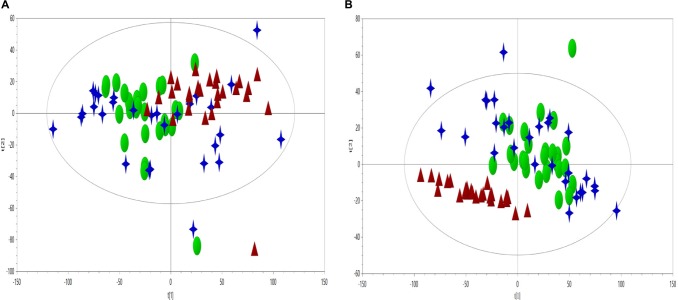
PCA scores plot (**A**, *R*^2^*X* = 0.636, *Q*^2^ = 0.560) and PLS-DA scores plot (**B**, *R*^2^*X* = 0.599, *Q*^2^ = 0.318, *R*^2^*Y* = 0.37) derived from NMR data to compare the metabolome of the control (triangles, red), CHD–BSS (circles, green) and CHD–PS (stars, blue).

Principal component analysis on plasma CPMG spectra were applied to observe the classification of CHD patients and controls. The first two principal components were plotted to present the distribution of the three groups (Figure 2).

As visually observed, the three groups were totally overlapped in the PCA scores plot, suggesting that the unsupervised PCA method could not extract useful information in the NMR CMPG data. PLS-DA was also used to show the classification of the three groups, as presented in Figure 2, and only the differences between the CHD and control groups could be observed. As for BSS and PS of  CHD patients, they were completely overlapped, and had no separation trend. PCA and PLS-DA on plasma NOESY and BPP-LED spectra, and model verification of PLS-DA on plasma CMPG spectra, BPP-LED spectra and NOESY spectra were shown in Supplemetary Figures S1–S5.

**FIGURE 3:**
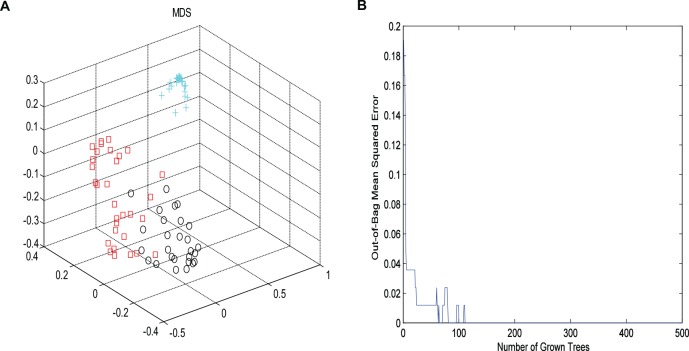
The MDS plot **(A)** for plasma profiles derived from NMR data for control (cross, blue), CHD–BSS (circles, black) and CHD–PS (squares, red). Plot of OOB error for RF classification of the three groups **(B)**.

Then, RF method was used to explore the underlying characteristics of this NMR data. 2000 trees were grown, during the trees growing, proximities were computed for the cases. Similar cases may fall into the same terminal node or derive from the same parent. In order to better present the differences of the samples, MDS was employed to map the proximity into a lower dimensional space. OOB estimate of error rate and fivefold cross validation were used to evaluate the stability of the forest tree model. It could be seen from Figure 3, the OOB error rate did not decrease with the number of trees constructed, the RF algorithm could avoid overfitting to a certain extent, and the area under the curve of ROC was 0.96. As shown in Figure 3A, the controls were far away from other two groups. Furthermore, an obvious distinction between CHD–BSS and CHD–PS was observed in the MDS plot. Figure 3B showed the OOB error rate.

It can be seen from Figure 3, the OOB error rate did not decrease with the number of trees constructed, the RF algorithm could avoid overfitting to a certain extent.

### Discovery of Plasma ^1^H-NMR Potential Biomarkers in CHD Patients and CHD–BSS Patients

**FIGURE 4:**
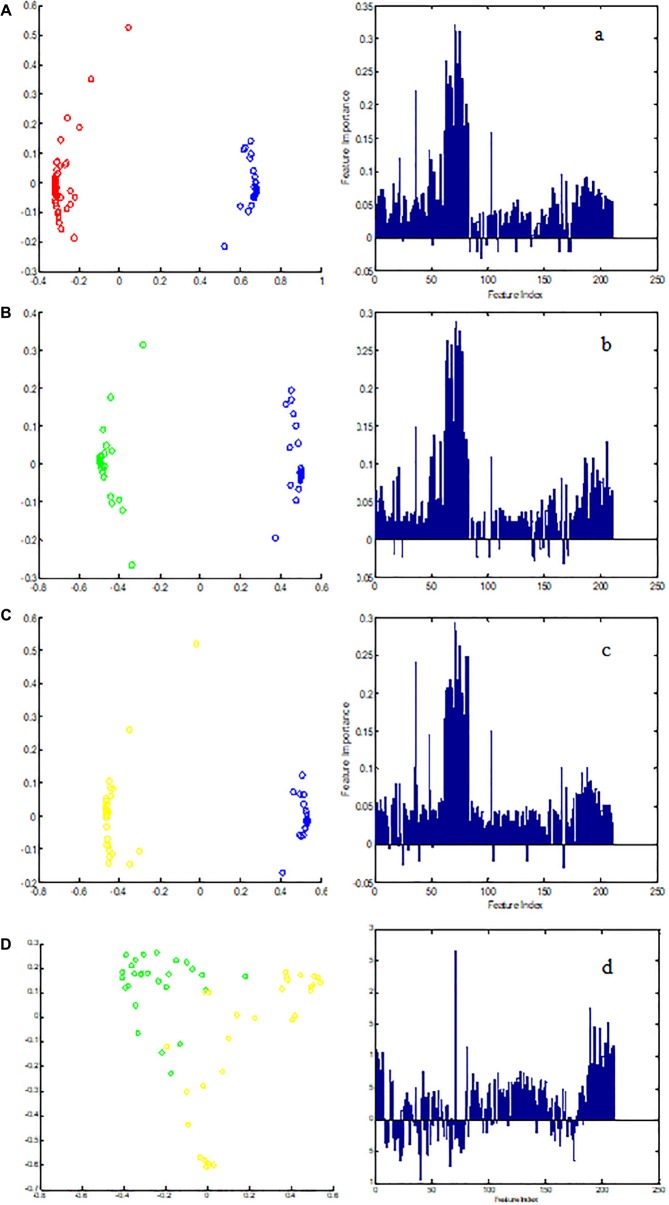
The MDS plot of **(A)** control (blue) and CHD (red), **(B)** control and CHD–BSS (green), **(C)** control and CHD–NBSS (yellow), and **(D)** CHD–BSS and CHD–NBSS. The VIM plot of **(a)** control and CHD, **(b)** control and CHD–BSS, **(c)** control and CHD–NBSS, and **(d)** CHD–BSS and CHD–NBSS obtained by random forest were shown in the right.

**FIGURE 5:**
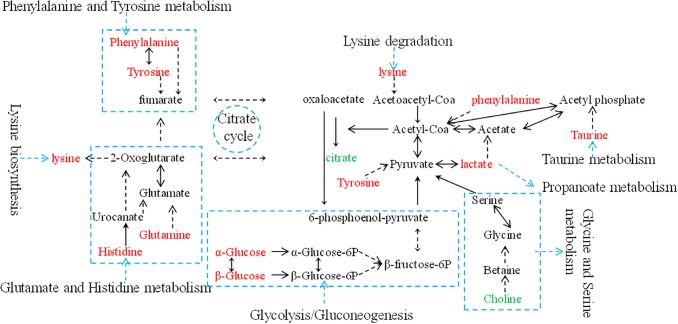
An overview of the metabolic pathway alterations related to CHD. Metabolite levels through color coding as follows: red, increase; green, decrease.

Metabolic biomarker discovery is an important aim of metabolomics studies. In model construction, the purpose of variable selection is to find the best combination of variables, which provide the best classification result (Huang et al., 2013). A measure of how each feature contributes to the prediction performance of RF can be calculated in the course of training, and its importance score (VIM) was obtained. VIM was used to measure the contribution of the variable to the classification. Figure 4 showed MDS plots and the variable importance. The feature importance was set to 0.1, from the bar plot of variable importance, we found that some metabolites make a contribution to the classification of controls and CHD samples (Figure 4a), the peak area quantitative of each spectrum were manually achieved by segmenting into 0.002 ppm in mestrenova, then *t*-test was implemented to test the significant of these metabolites and the result was summarized in Table 2 (*P* < 0.05). Finally, we obtained 12 characteristic metabolites, including lysine (1.40 ppm), glutamine (2.45 ppm), taurine (3.28 ppm), tyrosine (3.93 ppm), phenylalanine (3.96 ppm), histidine (4.0 ppm), lipid (2.0 ppm), citrate (2.55 ppm), choline (3.20 ppm, 4.05 ppm), lactate (4.11 ppm), α-Glucose (5.23 ppm, 3.39 ppm, 3.56 ppm, 3.71 ppm,3.83 ppm, 3.89 ppm), and β-Glucose (3.47 ppm, 3.72 ppm) related to the CHD patients. These metabolites involved in the key metabolic pathways are shown in Figure 5.

**TABLE 2:** The summaries of the metabolites that contributed to the clustering of CHD patients and angiography normal (controls) (*P* < 0.01).

**Important**	**Chemical**	**Potential**	**Peak area**	**Peak area in**	
**variables**	**shift (ppm)**	**biomarker**	**in controls**	**CHD patients**	***P***
22	1.403	Lysine	12.31 ± 9.82	39.60 ± 35.78	0.000
36	1.963	Lipid (CH_2_C=C)	59.58 ± 8.84	546.19 ± 440.76	0.000
48	2.443	Glutamine	69.39 ± 10.88	164.45 ± 99.36	0.000
50	2.523	Citrate	55.25 ± 10.79	35.83 ± 11.83	0.000
62	3.203	Choline	337.94 ± 55.40	208.86 ± 72.96	0.000
63	3.283	Taurine	61.80 ± 18.77	140.70 ± 38.35	0.000
64	3.323	α-glucose	8.26 ± 7.97	45.48 ± 19.54	0.000
66	3.403	α-glucose	85.09 ± 19.24	236.05 ± 126.66	0.000
67	3.443	β-glucose	111.78 ± 19.55	387.09 ± 201.84	0.000
68	3.483	β-glucose	134.09 ± 28.85	513.31 ± 292.54	0.000
69	3.523	α-glucose	132.77 ± 26.66	368.55 ± 210.79	0.000
70	3.563	α-glucose	180.07 ± 30.66	572.60 ± 332.92	0.000
71	3.643	Choline	3044.12 ± 833.95	537.74 ± 335.80	0.000
72	3.683	α-glucose	58.88 ± 41.69	709.10 ± 486.93	0.000
73	3.723	β-glucose	48.88 ± 25.24	290.72 ± 137.02	0.000
74	3.763	α-glucose	79.92 ± 17.81	260.75 ± 112.37	0.000
75	3.803	α-glucose	73.26 ± 14.69	652.03 ± 417.20	0.000
76	3.843	α-glucose	89.50 ± 20.28	241.12 ± 117.13	0.000
77	3.883	α-glucose	77.35 ± 14.27	197.34 ± 247.43	0.016
78	3.923	Tyrosine	107.86 ± 18.32	274.94 ± 117.29	0.000
79	3.963	phenylalanine	44.95 ± 14.34	143.55 ± 82.69	0.000
80	4.003	histidine	54.72 ± 14.74	106.16 ± 44.69	0.000
81	4.043	Choline	34.25 ± 15.40	253.45 ± 174.81	0.000
82	4.083	Lactate	58.42 ± 17.47	395.31 ± 295.24	0.000
103	5.243	α-glucose	−33.66 ± 29.03	58.58 ± 90.43	0.000

The variable importance measures of samples obtained by RF models revealed that in the plasma of CHD patients contained significantly higher levels of some amino acids, including lysine, glutamine, taurine, tyrosine, phenylalanine and histidine compared to the controls. Plasma lipid, lactate, α-glucose and β-glucose in CHD patients were higher when compared with controls (*P* < 0.01). However, citrate and choline of plasma from CHD patients were lower than those from angiography normal (controls) (*P* < 0.01).

In order to explore potential metabolite biomarkers of CHD–BSS patients, RF methodology was applied to extract important variables of the control group and CHD group. The feature importance was enhanced to 0.2 to obtain the most important variables. The variable importance measures of each two groups obtained by RF models were shown in Figure 4. The important variables (*P* < 0.01) of the each two groups were shown in Table 3, we found that 72/choline, 73/β-glucose, 74/α-glucose, 75/α-glucose, 77/α-glucose, 78/tyrosine could be the potential metabolite biomarkers of CHD–BSS patients.

**TABLE 3:** The important variables (*P* < 0.01) of the each two groups.

**Group**	**Important variables/metabolites**
Control-CHD	36/lipid, 63/taurine, 64/α-glucose, 67/β-glucose, 68/β-glucose, 71/choline, 72/choline, 73/β-glucose, 74/α-glucose, 75/α-glucose, 77/α-glucose, 78/tyrosine, 81/choline
Control-BSS	63/taurine, 64/α-glucose, 67/β-glucose, 68/β-glucose, 71/choline, 72/choline, 73/β-glucose, 74/α-glucose, 75/α-glucose, 77/α-glucose, 78/tyrosine
Control-PS	36/lipid, 64/α-glucose, 67/β-glucose, 68/β-glucose, 71/choline, 72/choline, 73/β-glucose, 75/α-glucose, 81/choline, 82/lactate
BSS–PS	71/choline

## Discussion

The use of the analytical techniques, ^1^H-NMR, was feasible to study the metabonomic differences between angiography normal and CHD patients. ^1^H-NMR analysis enabled the identification of a total of 12 metabolites as contributors to the discrimination of controls and CHD patients. We found that the potential biomarkers were principally correlated to lipid metabolism dysfunction, energy metabolism dysfunction, amino acid dysfunction and glucose metabolism dysfunction in the pathological development of CHD. In our previous studies using LC-MS, we found 27 potential biomarkers which were principally involved in arachidonic acid metabolism, amino acid metabolism, purine metabolism, pyrimidine metabolism, steroid biosynthesis and linoleic acid metabolism that discriminated CHD patients from healthy controls. In those potential biomarkers, citric acid and phenylalanine were found in the current research with NMR technology. Integration of several metabonomics technologies could provide more comprehensive biomarker information.

Choline is an important methyl donor, precursor of acetylcholine, and it is needed for lipid metabolism (Ueland, 2011). Choline can be converted to betaine in a two-step enzymatic reaction occurring mainly in the mitochondria of liver cells. Choline or betaine may be important for lowering plasma homocysteine concentrations (Steenge et al., 2003), which is associated with increased risk of cardiovascular disease (CVD) (Homocysteine Studies Collaboration, 2002). LC-HRMS method was applied to measure the plasma concentrations of choline in a cohort of 339 patients undergoing coronary angiography for the evaluation of suspected coronary artery disease, the results show plasma levels of choline are significantly lower in patients with a history of acute myocardial infarction as compared to those without such history (Mueller et al., 2015). Brindle et al. (2002) have shown that choline contributed most strongly to the discrimination of the CHD and control group. Choline (Oliver and Martin, 2010) are emerging biomarkers in acute coronary syndrome. Our study demonstrated that plasma choline levels in patients with CHD remarkably decreased, suggesting the relationship between choline and CHD risk.

Abnormal metabolism of the lactate and citrate were the indications of energy metabolism abnormality. Lactate, which is an end product of glucose anaerobic glycolysis, is reportedly a useful indicator for ischemia according to the clinical and scientific studies. It has been found that lactate was remarkably increased in plasma of patients with CHD in this study which suggested that glycolysis was activated by myocardial ischemia and hypoxia. Previous studies (Barderas et al., 2011; Rowland et al., 2013) have shown an obvious lactate increased in CHD patients. The citric acid cycle plays a central role in oxidative phosphorylation in the myocardium. In the setting of acute ischemia, preservation of citric acid cycle intermediates becomes of paramount importance to defend ATP production. Sabatine et al. (2005) have demonstrated highly statistically significant changes in circulating levels of metabolites belonging to the citric acid pathway, including citrate. In our study, the citrate level was also found lower in the CHD group than in the control group.

In the metabolic processes of the amino acids, plasma lysine, glutamine, taurine, tyrosine, phenylalanine and histidine of the CHD patients had increased significantly in this study, especially taurine and tyrosine. It has been found in several metabolic studies that amino acid metabolism is frequently abnormal in patients with CHD. The surplus amino acids provided adequate raw materials for the synthesis of lipid, thus abnormal lipid metabolism of CHD patients had been aggravated at the same time.

Our study found that the level of α-glucose, β-glucose in the plasma of CHD patients was higher than that in the control group. Glucose, as a short-term marker for glycemic control (Dungan, 2008), could indicate that significant disorders of glucose metabolism were happened in CHD patients, and CHD individuals at higher risk for developing diabetes or insulin resistance.

Furthermore, this study showed a clear metabonomic difference between CHD–BSS group and CHD–PS group (Figure 4), indicating CHD–BSS and CHD–PS were different metabolism patterns. Choline, β-glucose, α-glucose and tyrosine were considered as potential biomarkers of CHD–BSS in this study. Choline can prevent CVD by preventing the deposition of TC in the inner wall of blood vessels and improving the absorption and utilization of fat. A proteome study (Zhao et al., 2008) found that there was lipid metabolism disorder in patients with CHD–BSS. Apolipoproteins in plasma of patients with CHD–BSS decreased. Apolipoproteins A IV is involved in the reverse transport of cholesterol. In our previous studies, amino acids, fatty acids, purine and so on were selected as a panel of candidate biomarkers of CHD–BSS based on GC-MS (Wei et al., 2013) and LC-MS (Zhao et al., 2014) technology, integration of several technologies could provide more comprehensive classification and biomarker information. It suggested that the potential biomarkers revealed by the three techniques were supplementary. In addition, PCA and PLS-DA method could not extract useful information in the NMR CMPG data for pattern-recognition analyses of biological samples. The RF method could be used to explore more the underlying characteristics of biological samples in this study. Therefore, it is important to develop more efficient pattern recognition approach for the analysis of complex metabolomics data. CHD–BSS is not exist alone in the clinics, so one limitation is that CHD–BSS group included in the study is also mixed with other syndromes, such as qi deficiency, yin deficiency, etc., we will enroll patients of BSS with different diseases and adopt targeted LC-MS/MS analysis to verify the potential markers of BSS in future studies.

## Conclusion

Our study demonstrated a clear metabonomic difference between CHD–BSS group and CHD–PS group, indicating CHD–BSS and CHD–PS were different metabolism patterns. Choline, β-glucose, α-glucose and tyrosine were considered as potential biomarkers of CHD–BSS. It is also worth noting that RF and MDS could be used to explore more the underlying characteristics of biological samples in this study, and RF could effectively mine the pattern information hidden in the complex metabolomics data.

## Data Availability

All datasets generated for this study are included in the manuscript and the Supplementary Files.

## Ethics Statement

This study was carried out in accordance with the recommendations of “The IRB of Xiangya Hospital, Central South University” with written informed consent from all subjects. All subjects gave written informed consent in accordance with the Declaration of Helsinki. The protocol was approved by the “The IRB of Xiangya Hospital, Central South University.”

## Author Contributions

D-SW conceived and designed the research. L-LZ and W-BW performed the following research projects. X-JQ, R-ML, and D-SW contributed toward the identification of disease and ZHENG types. L-LZ conducted data pre-processing of the NMR spectra, data analysis, and drafted the manuscript. W-BW and D-SW contributed toward the results validation. All authors revised the manuscript.

## Conflict of Interest Statement

The authors declare that the research was conducted in the absence of any commercial or financial relationships that could be construed as a potential conflict of interest.
