# Supplementary material for: NMR Metabolomics and Random Forests Models to Identify Potential Plasma Biomarkers of Blood Stasis Syndrome With Coronary Heart Disease Patients
Source: Front Physiol. 2019 Sep 4;10:1109. doi: 10.3389/fphys.2019.01109 (PMC6738169; doi:10.3389/fphys.2019.01109)
Supplement: Supplementary file 1 [file Table_1.DOCX]

PCA on plasma NOESY spectra were applied to observe the classification of CHD patients and controls. The first two principal components were plotted to present the distribution of the three groups (FIG. 1).

FIG. 1 PCA scores plot (left, R^2^X=0.992, Q^2^=0.976) and PLS-DA scores plot (right, R^2^X=0.782, Q^2^=0.227, R^2^Y=0.37) derived from NMR data to compare the metabolome of the control (red), CHD-BSS (green) and CHD-PS (blue).

FIG.2 Model verification of PLS-DA on plasma NOESY spectra

The replacement test of the PLS-DA on plasma NOESY spectra model was carried out 200 times, As shown on Fig 2, the values of R2 and Q2 on the left were lower than the values of R2 and Q2 on the right, and the intercept of Q2 regression line was negative, which indicates that the PLS-DA model did not have the phenomenon of fitting and had a good prediction ability.

PCA on plasma BPP-LED spectra were applied to observe the classification of CHD patients and controls. The first two principal components were plotted to present the distribution of the three groups (FIG. 3).

FIG. 3 PCA scores plot (left, R^2^X=0.99, Q^2^=0.975) and PLS-DA scores plot (right, R^2^X=0.779, Q^2^=0.033, R^2^Y=0.095) derived from NMR data to compare the metabolome of the control (red), CHD-BSS (green) and CHD-PS (blue).

FIG.4 Model verification of PLS-DA on plasma BPP-LED spectra

The replacement test of the PLS-DA on plasma BPP-LED spectra model was carried out 200 times, As shown on Fig 4, the values of R2 and Q2 on the left were lower than the values of R2 and Q2 on the right, and the intercept of Q2 regression line was negative, which indicates that the PLS-DA model did not have the phenomenon of fitting and had a good prediction ability.

FIG. 5 Model verification of PLS-DA on plasma CMPG spectra

The replacement test of the PLS-DA on plasma CMPG spectra model was carried out 200 times, As shown on Fig 5, the values of R2 and Q2 on the left were lower than the values of R2 and Q2 on the right, and the intercept of Q2 regression line was negative, which indicates that the PLS-DA model did not have the phenomenon of fitting and had a good prediction ability.
